# Exposure to intermittent hemodialysis and renal recovery after acute kidney injury: a systematic review

**DOI:** 10.1186/cc10975

**Published:** 2012-03-20

**Authors:** A Schneider, M Bagshaw, NJ Glassford, R Bellomo

**Affiliations:** 1 Austin Health, Heidelberg, Australia; 2 University of Alberta, Edmonton, Canada

## Introduction

Renal replacement therapy (RRT) in critically ill patients can be applied in a continuous (CRRT) or intermittent (IRRT) fashion. To date, there is no systematic comparison on the impact of these two modalities on renal recovery after an episode of acute kidney injury (AKI). We sought to compare the rates of renal recovery with RRT independence between CRRT and IRRT as an initial modality for RRT in AKI.

## Methods

We searched MEDLINE and EMBASE. We retrieved all studies published between 2000 and 2010 that report original data on renal recovery to RRT dependence after AKI in adults. Authors of studies with incomplete data were contacted. Search date: January 2011. Two authors independently assessed the trial quality and extracted data. Pooled analyses were performed and a chi-square test performed. Sensitivity analyses were performed after stratification by premorbid chronic kidney disease, number of centers, type of study and illness severity index. In a subsequent analysis we pooled the studies according to the percentage of patients exposed to IHD into low-exposure (<50% of patients exposed) or high-exposure (>50% patients exposed).

## Results

We identified 50 studies (14,796 patients). Overall, as compared with those that received IRRT as an initial modality (IRRT group), those that received CRRT (CRRT group) had higher average illness severity scores (mean APACHE III equivalent 88 vs. 72, *P *< 0.01) and higher in-hospital mortality (57.7% vs. 37.9%, *P *< 0.0001). When reported at 28 days after initiation of RRT (outcome reported in 25 studies), 19.4% of survivors were RRT dependent in the CRRT group versus 26.9% in the IRRT group (*P *= 0.004). At hospital discharge (reported in 26 studies), RRT dependence was present in 10.9% of the CRRT group versus 20.8% in the IRRT group (*P *< 0.0001). At day 90 (reported in 22 studies), RRT dependence was 7.8% in the CRRT group versus 36.1% in the IRRT group (*P *< 0.0001). The sensitivity analyses confirmed these findings in all subgroups. The rates of RRT dependency in the low-exposure group and the high-exposure group at days 28, 90 and hospital discharge were 19.6%, 8.8% and 12.4% versus 43.2%, 26.8% and 14.0% respectively (all *P *< 0.0001, except for hospital discharge *P *= NS).

## Conclusion

The preponderance of the available evidence suggests that CRRT is associated with a higher rate of renal recovery in AKI survivors compared with IRRT.

